# Impact of amygdala functional connectivity on cognitive impairment in Alzheimer’s disease

**DOI:** 10.1007/s10072-025-08091-0

**Published:** 2025-03-08

**Authors:** Ani Kicik, Elif Kurt, Emre Hari, Çiğdem Ulasoglu-Yildiz, Hakan Gurvit, Tamer Demiralp

**Affiliations:** 1 Department of Physiology, Faculty of Medicine, Demiroglu Bilim University, Istanbul, 34394 Turkey; 2 Department of Neuroscience, Aziz Sancar Institute of Experimental Medicine, Istanbul University, Istanbul, 34093 Turkey; 3 Department of Physiology, Istanbul Faculty of Medicine, Istanbul University, Istanbul, 34093 Turkey; 4 Graduate School of Health Sciences, Istanbul University, Istanbul, 34216 Turkey; 5 Behavioral Neurology and Movement Disorders Unit, Department of Neurology, Istanbul Faculty of Medicine, Istanbul University, Istanbul, 34093 Turkey; 6 Hulusi Behcet Life Sciences Research Laboratory, Neuroimaging Unit, Istanbul University, Istanbul, 34093 Turkey

**Keywords:** Alzheimer’s disease, Magnetic resonance imaging, Functional connectivity, Amygdala, Cognitive impairment

## Abstract

The functional connectivity (FC) of the amygdala in Alzheimer’s disease (AD) and its relationship to cognitive impairment is still not well established. Thus, we examined resting-state FC changes in the amygdala among 21 patients with AD dementia (ADD) and 34 individuals with amnestic mild cognitive impairment (aMCI), compared to 33 individuals with subjective cognitive impairment (SCI), to provide insights into the association between amygdala FC and cognitive decline in different clinical stages of Alzheimer’s disease. We conducted seed-to-voxel FC analysis, focused on two cognitive functions, episodic memory, and face recognition, and examined the correlations between changes in FC of the amygdala and cognitive test scores. We demonstrated that the left amygdala exhibits progressive disruption in FC, especially with the frontal regions in aMCI and ADD. We further identified that this disrupted FC in the left amygdala showed significant positive correlations with cognitive test scores from the MCI stage onward. Our results indicate that FC changes in the left amygdala may serve as an early marker of AD and this FC pattern of amygdala influence detrimentally affects episodic memory and face recognition functions. These findings highlight that the amygdala may be a critical anatomical region for detecting the early stages of AD.

## Introduction

Alzheimer’s disease (AD) is the most prevalent neurodegenerative disorder, typically characterized by the impairment in memory initially and in other cognitive functions subsequently. The neuropathological correlates of the typical memory impairment in AD echo in Braak and Braak’s staging of neurofibrillary tangle (NFT) pathology, which states that the entorhinal cortex (ERC), transentorhinal cortex (TEC), and hippocampus, the core limbic structures of the episodic memory neural network are the earliest to be invaded by the NFTs and accordingly, much of the research has focused on these areas [1, 2]. However, recent imaging studies increasingly indicate that the amygdala may play an early role in AD, with evidence showing that it is affected by NFT pathology in the initial stages [3–5]. Several tau-PET studies have shown that amyloid-positive individual’s exhibit elevated tau accumulation in the amygdala, even in the absence of cognitive decline, suggesting that pathological changes in the amygdala in AD may begin prior to the onset of cognitive symptoms [3, 6, 7]. Furthermore, recently many structural MRI studies have demonstrated that decreased amygdala volume is associated with memory impairtment, as well as global cognitive decline in AD [8–10].

Regarding the role of the amygdala in the early stages of AD, recent findings suggest that amygdala imaging measures have the potential to serve as a biomarker for clinical applications [2]. Nonetheless, studies investigating functional connectivity (FC) changes in the amygdala and their relationship with disease severity and cognitive decline are limited. Further research is required to clarify the role of the amygdala in AD and to associate it with cognitive function and the progression of the disease. For this purpose, we investigated resting-state FC changes in the amygdala in the patients with AD dementia (ADD) and amnestic mild cognitive impairment (aMCI) compared to individuals with subjective cognitive impairment (SCI).

Memory impairment is a hallmark symptom observed in AD; however, the amygdala’s critical role in emotional processing has led to more extensive research on its association with emotional disturbances in the Alzheimer’s disease continuum than on its involvement in memory impairment [2, 11]. In addition to its role in emotional regulation and its contribution to memory processes, such as encoding and consolidation, there are several studies suggesting that the amygdala has a modulatory effect on face perception and plays an important role in the processing of face recognition [12–14]. However, the relationship between changes in FC of the amygdala and facial recognition impairment in AD has not been well studied. For these reasons, by focusing on these two cognitive functions (i.e., episodic memory and facial recognition), we examined the correlations between FC changes in the amygdala and the Free and Cued Selective Reminding Test (FCSRT) and the Benton’s Facial Recognition Test (BFRT) scores to evaluate the impact of FC changes of the amygdala on cognitive impairments in aMCI and ADD patients. We hypothesized that there would be progressive disruptions in the FC of the amygdala from the early stages of AD, and that these disruptions would be associated with deficits in episodic memory and facial recognition functions.

## Materials and methods

### Participants

The study comprised 21 individuals diagnosed with mild ADD, 34 with amnestic MCI (aMCI), and 33 with SCI, all of whom were diagnosed at the Department of Neurology, Behavioral Neurology and Movement Disorders Unit at Istanbul University, Istanbul Faculty of Medicine. All participants underwent clinical and neuropsychological assessments, including the Clinical Dementia Rating scale (CDR), Mini-Mental State Examination (MMSE), FCSRT, and BFRT. Since cerebrospinal fluid biomarker data were not available for the entire participants the diagnostic classification of the three groups was based on clinical diagnoses. A score of ≤ 24 on the FCSRT - Total Free Recall (FCSRT-TFR) was used as the cutoff criterion for identifying objective memory impairment [15]. The ADD group patients fulfilled the clinical criteria established by the National Institute on Aging and Alzheimer’s Association (NIA-AA) for very mild or mild typical probable ADD with amnestic presentation, had a CDR score of 0.5 (and CDR Sum of the Boxes score [CDR-SOB] > 1) or 1, and did not present with any neuropsychiatric diagnoses aside from dementia [16]. The aMCI group consisted of patients diagnosed with amnestic MCI based on Petersen’s criteria [17], meeting the following conditions: (1) a Clinical Dementia Rating (CDR) score of 0.5 and a CDR-SOB≤1; (2) objective memory impairment, isolated or prominent among multiple domains, demonstrated by an FCSRT-TFR score of ≤ 24 [15]; (3) had no other neuropsychiatric diagnosis. Participants were identified as having SCI according to the following criteria: (1) subjective memory complaints; (2) CDR = 0, (3) FCSRT-TFR > 24 [18].

The exclusion criteria were: (1) significant psychiatric diseases such as major depression and schizophrenia; (2) chronic systemic diseasemay affect cognition;3) neurologic comorbidity; 4) a history of stroke and head trauma accompanied by loss of cognition; 5) significant structural brain abnormalities other than mild white-matter FLAIR hyperintensities observed in the clinical MRI examinations.

### MRI acquisition

MR images were obtained using a 3T MRI scanner (Achieva, Philips, The Netherlands), equipped with a 32-channel SENSE head coil at the Istanbul University Hulusi Behçet Life Sciences Research Laboratory Neuroimaging Unit. Resting-state functional images were obtained using an echo-planar imaging (EPI) sequence in the axial plane with the following parameters: repetition time (TR) / echo time (TE): 3000 / 30 ms, field of view (FOV) = 212 × 199 mm, voxel size: 3.31 × 3.31 × 3. 31 mm, acquisition matrix size: 64 × 59, number of slices = 48, flip angle = 80°. High-resolution T1-weighted structural images were acquired using a 3D turbo field echo sequence (TFE) with the following parameters: TR/TE: 8.3 / 3.8 ms, FOV: 220 × 240 mm, 180 axial slices (with no gap), acquisition matrix: 252 × 227, 1 mm^3^ isotropic voxels, flip angle: 8°.

### Functional MRI analysis

Functional MRI data were preprocessed using the standard preprocessing pipeline in the CONN functional connectivity toolbox version 18.b based on Statistical Parametric Mapping version 12 (SPM 12, http://www.fil.ion.ucl.ac.uk/spm/) [19]. First, functional images were realigned to the first volume to correct for head motion. The Artifact Detection Tools (ART) toolbox was used to identify outlier volumes [20]. Outlier volumes were determined based on global-signal (GS) values with a threshold set at 5 standard deviations and frame-wise displacement (FD) values with threshold set as ≥ 0.9 mm difference in composite motion of an image from the previous scan. In the next step, functional data coregistered to the structural images and these structural images were then segmented into white matter (WM), gray matter (GM), and cerebrospinal fluid (CSF). The transformation matrix obtained during the normalization of the structural data to the Montreal Neurological Institute (MNI) standard space was used for the spatial normalization of the functional images. Finally, the functional data were resampled to an isotropic voxel size of 2 mm^3^ and smoothed using a Gaussian kernel of 8-mm full width at half maximum. The denoising procedure was applied to functional data to eliminate noise factors due to movement and physiological effects. The anatomical component-based noise correction (aCompCor) method was utilized to estimate noise components from WM and CSF signals. For the denoising process, these estimated components, along with motion parameters, and ART-based scrubbing parameters were regressed out. Finally, a band-pass filter of 0.01–0.1 Hz was applied to the functional images, followed by linear detrending.

Seed-to-voxel functional connectivity analyses were performed using the CONN toolbox. FSL Harvard-Oxford atlas was used to define bilateral amygdala seeds. For the FC analyses, first, subject-level FC analysis was performed by computing the bivariate correlation coefficients between the average time courses of each seed and the time courses of all other voxels in the brain. Second-level analyses were conducted on Pearson’s correlation coefficients converted to Z-scores with Fisher’s Z transformation. FC differences were compared among the three groups using analysis of variance (ANOVA). Two-sample t-tests were used to determine the FC changes in the pairwise comparisons. Functional connectivity results for all analyses were thresholded with a voxel level threshold set at *p* < 0.001 (uncorrected) and a cluster level threshold set at *p* < 0.05 corrected for family-wise error (FWE).

### Statistical analysis

Statistical analyses of demographic and clinical variables were performed with IBM SPSS 25. Statistical significance was accepted as *p* < 0.05. Pearson’s chi-squared test was used to compare gender. One-way ANOVA test was used to analyze normally distributed data and post hoc tests were performed by applying Bonferroni correction. To compare not normally distributed data, the Kruskal-Wallis test was used. For the multiple comparisons, the Mann–Whitney U test was used and *p* < 0.017 was considered statistically significant. The Spearman’s correlation test was used to analyze the relationship between the values of the functional connection of the amygdala and neuropsychological test scores (MMSE, FCSRT, BFRT).

As mentioned above, the FCSRT-TFR score was used for the diagnostic classification of the aMCI and SCI groups. Other FCSRT subscores were used in the correlation analysis, namely the FCSRT - Total Recall (FCSRT-TR), FCSRT - Delayed Free Recall (FCSRT-DFR), and FCSRT - Delayed Total Recall (FCSRT-DTR).

## Results

### Demographic and neuropsychological characteristics of participants

No significant differences were observed among the ADD, aMCI, and SCI groups with respect to age, education, and gender. There were significant differences in FCSRT subscores, MMSE, and, BFRT scores among the three groups (Table 1). Post hoc analyses for MMSE showed that the scores of the ADD group were lower than those of the aMCI (*p* < 0.001) and SCI groups (*p* < 0.001), and the aMCI group’s scores were lower compared to the SCI group (*p* = 0.005). Post hoc analyses for FCSRT-TFR, FCSRT-TR, and FCSRT-DTR showed that the scores of the ADD group were lower than those of the aMCI (*p* < 0.001) and the SCI groups (*p* < 0.001), and aMCI group’s scores were lower compared to the SCI group (*p* < 0.001). Post hoc analyses for FCSRT-DFR showed that the scores of the ADD group were lower than those of the aMCI (*p* = 0.001) and SCI groups (*p* < 0.001), and the aMCI group’s scores were lower compared to the SCI group (*p* < 0.001). Post hoc analyses for BFRT showed that the ADD group’s score was lower than those of the aMCI (*p* = 0.011) and SCI (*p* < 0.001) groups, but BFRT scores did not differ between the aMCI and SCI groups (*p* = 0.057). The demographic and clinical characteristics are shown in Table 1.

**Table 1:** The demographic and clinical characteristics of the ADD, aMCI and SCI groups

	ADD (*n* = 21)	aMCI (*n* = 34)	SCI (*n* = 33)	Statistics, *P* value
	Mean (SD)	Mean (SD)	Mean (SD)	
Gender (F/M)	10/11	13/21	22/11	χ^2^ = 5.55^a^, 0.062
Age (year)	67.43 (9.93)	63.79 (7.17)	63.12 (8.01)	*F* = 1.92^b^, 0.153
Education (year)	10.86 (4.62)	10.94 (4.93)	13.12 (4.72)	χ^2^ = 2.91^c^, 0.233
MMSE	23.33 (3.79)	27.91 (1.71)	28.94 (1.39)	χ^2^ = 37.31^c^, < 0.001
FCSRT-TFR	8.14 (7.25)	18.35 (4.38)	30.85 (4.42)	χ^2^ = 68.95^c^, < 0.001
FCSRT-TR	22.65 (11.39)	36.76 (6.04)	45 (3.20)	χ^2^ = 57.45^c^, < 0.001
FCSRT-DFR	2.80 (2.81)	6.24 (3.34)	11.88 (2.43)	χ^2^ = 55.21^c^, < 0.001
FCSRT-DTR	7.75 (4.77)	12.56 (2.51)	15.31 (0.99)	χ^2^ = 47.74^c^, < 0.001
BFRT	42.30 (4.01)	45.61 (3.95)	47.91 (3.79)	*F* = 12.85^b^, < 0.001

^a^Pearson’s chi-squared test

^b^One-way ANOVA test

^c^Kruskal-Wallis H test

ADD: Alzheimer’s disease dementia; aMCI: Amnestic mild cognitive impairment; SCI: Subjective cognitive impairment; SD: Standard deviation; MMSE: Mini-mental state exam; FCSRT-TFR: Free and Cued Selective Reminding Test- Total Free Recall; FCSRT-TR: FCSRT- Total Recall; FCSRT-DFR: FCSRT - Delayed Free Recall; and FCSRT-DTR: FCSRT - Delayed Total Recall; BFRT: Benton’s Facial Recognition Test.

### Functional connectivity results

**Fig. 1:**
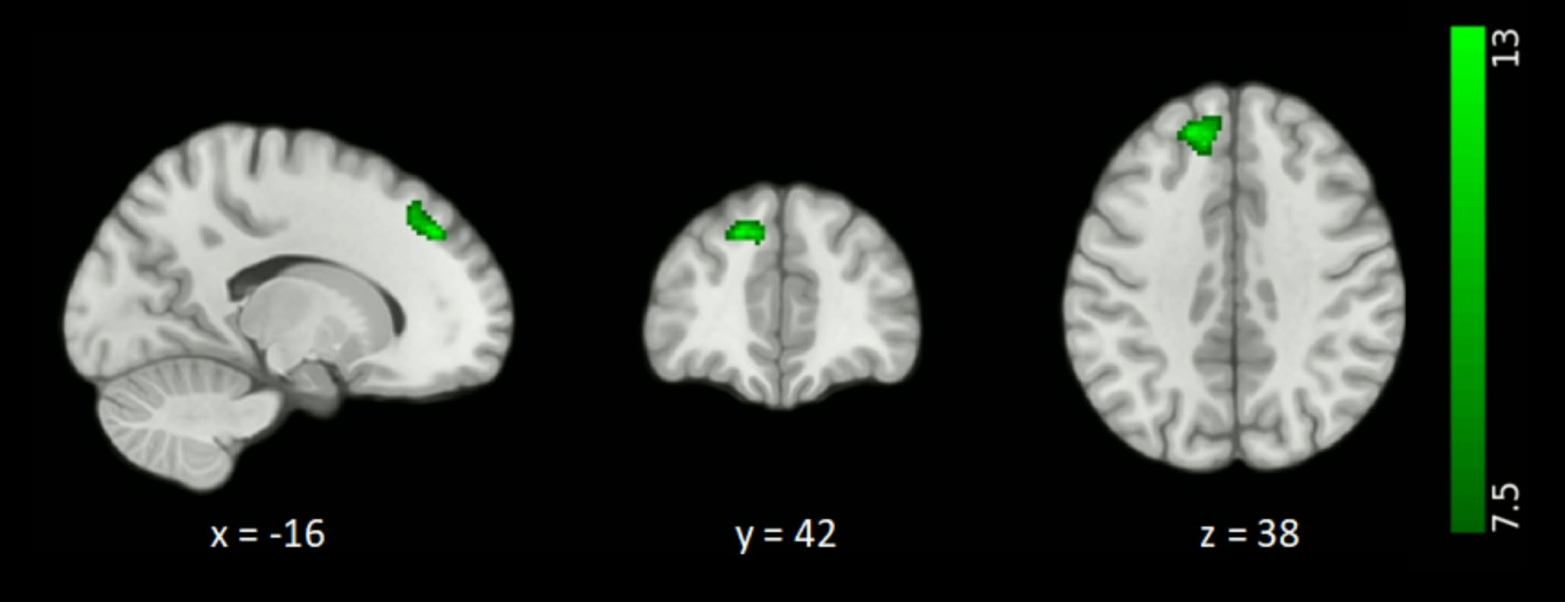
Functional connectivity differences among the ADD, aMCI and SCI groups for the left amygdala. The FC analysis was thresholded with a voxel level threshold set at *p* < 0.001 (uncorrected) and *p*FWE < 0.05 at the cluster level

Seed-to-voxel analyses revealed significant FC differences among the ADD, aMCI and SCI groups for the left amygdala. The left amygdala showed significantly decreased FC with the left superior frontal gyrus and medial frontal gyrus among the three groups (Fig. 1, Table 2), while no significant FC difference was obtained for the right amygdala.

**Table 2:** Functional connectivity differences between the groups for the left amygdala (voxel level threshold of *p* < 0.001 (uncorrected), cluster level *p*FWE-corrected threshold of *p* < 0.05)

Contrast	Regions showing connectivity changes	Cluster Size	Peak MNI coordinate	*p*FWE
ANOVA among the 3 groups	L-SFG, MFG	220	-16 42 38	0,03115
ADD < SCI	L-SFG, L-MidFG	783	-16 42 38	0,00006
	L-PreCG, L-PostCG	284	-34 -18 56	0,02271
	L-Fusiform gyrus	258	-36 -38 -18	0,03320
	L-IFG	238	-34 14 26	0,04476
aMCI < SCI	L-SFG, MFG	275	-4 62 14	0,03125

L: Left; SFG: Superior frontal gyrus; MFG: Medial frontal gyrus; MidFG: Middle frontal gyrus; PreCG: Precentral gyrus; PostCG: Postcentral gyrus; IFG: Inferior frontal gyrus; ADD: Alzheimer’s disease dementia; aMCI: Amnestic mild cognitive impairment; SCI: Subjective cognitive impairment; FWE: Family-wise error; MNI: Montreal Neurological Institute

**Fig. 2:**
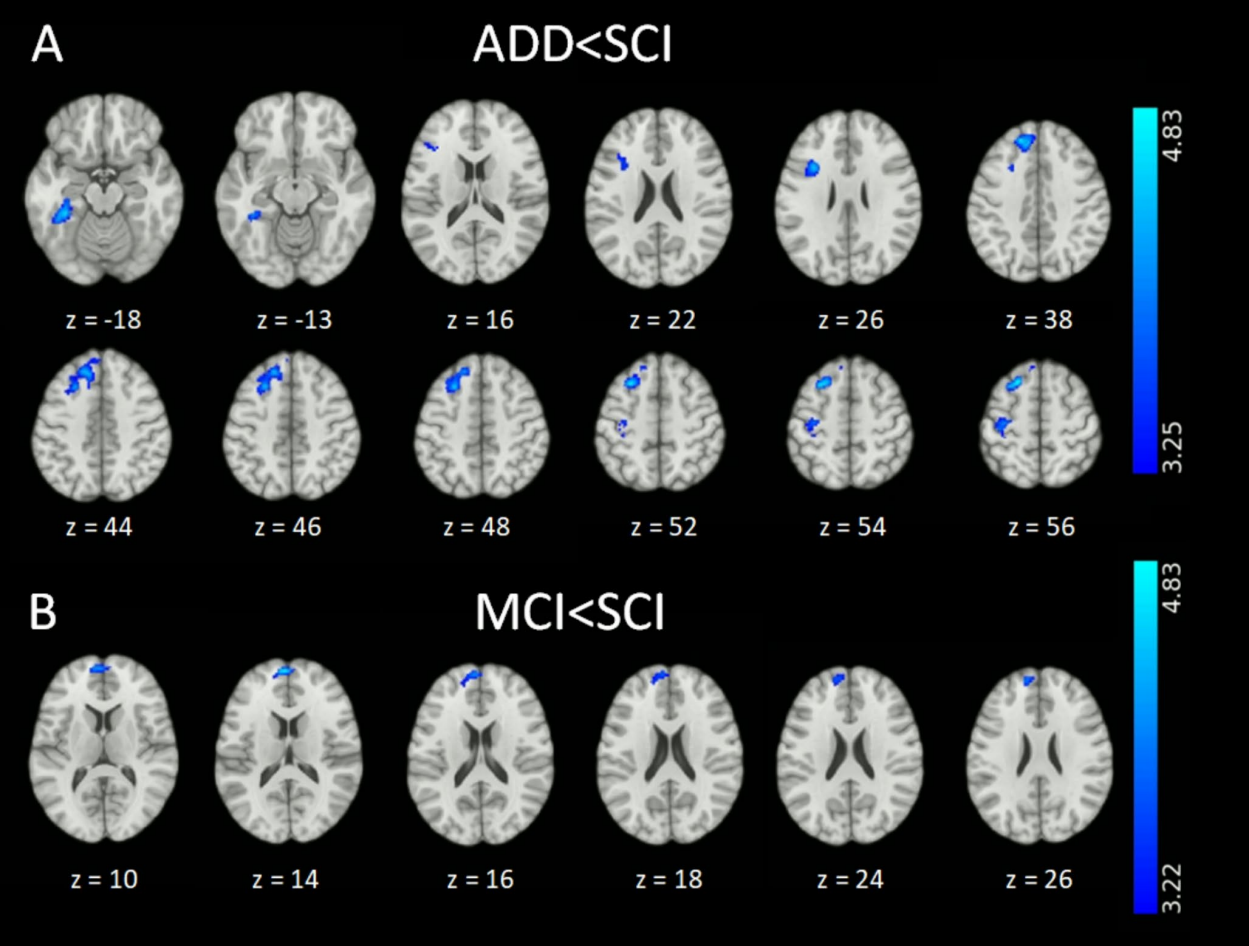
Functional connectivity differences for the left amygdala. (**A**) Regions that showed decreased functional connectivity in the ADD group compared to the SCI group. (**B**) Regions that showed decreased functional connectivity in the aMCI group compared to the SCI group. The threshold for pairwise comparisons was set to *p* < 0.001 (uncorrected) at the voxel level and *p*FWE < 0.05 at the cluster level

Post-hoc pairwise group comparisons revealed significantly decreased FC of the left amygdala with four clusters corresponding to several regions in the ADD compared to the SCI group. These regions were the left superior frontal gyrus, medial frontal gyrus, left inferior frontal gyrus, left middle frontal gyrus, left fusiform gyrus, and left precentral and postcentral gyri (Fig. 2, Table 2). Comparison between the aMCI and SCI groups revealed decreased FC of the left amygdala with a cluster corresponding to the left superior frontal gyrus and medial frontal gyrus (Fig. 2, Table 2). We did not find any significant differences in the FC of the left amygdala between the ADD and aMCI groups.

### Relationship between the functional connections of the amygdala and cognitive variables

The reduced FCs of the left amygdala identified in the pair-wise group comparisons were tested for correlations with FCSRT subscores (FCSRT-TFR, FCSRT-TR, FCSRT-DFR, and FCSRT-DTR), MMSE and, BFRT scores. For the clusters that revealed significant differences between the ADD and SCI groups, the reduced FC of the left amygdala with brain regions, including the left superior and middle frontal gyri, and left fusiform gyrus, showed significant positive correlations with all FCSRT subscores and MMSE scores (Table 3). Additionally, a significant positive correlation was also found between the BFRT scores and the left amygdala FC to the left fusiform gyrus (Table 3). For the significant differing clusters between the aMCI and SCI groups, FCSRT-TFR was positively correlated with the left amygdala FC with the medial frontal and left superior frontal regions (Table 3), but no significant FC correlations were present for the MMSE and BFRT scores.

**Table 3:** Correlations between the functional connections of the left amygdala and cognitive variables (MMSE, BFRT and FCSRT subscores) in the ADD, aMCI and SCI groups

	Brain Region	Brain Region	MMSE		FCSRT-TFR		FCSRT-TR		FCSRT-DFR		FCSRT-DTR		BFRT	
			*R*	*P*	*R*	*P*	*R*	*P*	*R*	*P*	*R*	*P*	*R*	*P*
ADD and SCI	Left Amygdala	Left superior, middle and medial frontal gyri	0.372	0.006	0.458	< 0.001	0.433	0.001	0.545	< 0.001	0.470	< 0.001	0.152	0.278
		Left precentral gyrus and postcentral gyri	0.330	0.015	0.508	< 0.001	0.406	0.003	0.520	< 0.001	0.435	0.001	0.295	0.032
		Left fusiform gyrus	0.412	0.002	0.562	< 0.001	0.397	0.003	0.455	0.001	0.490	< 0.001	0.399	0.003
		Left inferior and middle frontal gyri	0.272	0.046	0.439	0.001	0.390	0.004	0.417	0.002	0.416	0.002	0.269	0.052
aMCI and SCI	Left Amygdala	Left superior and medial frontal gyri	0.184	0.137	0.485	< 0.001	0.403	0.001	0.386	0.001	0.313	0.011	0.116	0.352

ADD: Alzheimer’s disease dementia; aMCI: Amnestic mild cognitive impairment; SCI: Subjective cognitive impairment; MMSE: Mini-mental state exam; FCSRT-TFR: Free and Cued Selective Reminding Test- Total Free Recall; FCSRT-TR: FCSRT- Total Recall; FCSRT-DFR: FCSRT - Delayed Free Recall; and FCSRT-DTR: FCSRT - Delayed Total Recall; BFRT: Benton Face Recognition Test.

## Discussion

In this study, we investigated functional connectivity differences of the amygdala in the ADD, aMCI, and SCI groups. We found that the ADD group had decreased FC between the left amygdala and the left superior and inferior frontal gyri, the medial frontal gyrus, the left precentral and postcentral gyri, and the left fusiform gyrus compared to the SCI group. In the aMCI group, we observed a reduction in FC between the left amygdala and similar frontal regions the including left superior and medial frontal gyri compared to the SCI group. There are few studies that show FC alterations of the amygdala in AD and MCI groups [21–23]. An fMRI study has demonstrated decreased FC between the amygdala and various brain regions including hippocampus, superior temporal gyrus (STG), precentral gyrus, inferior/superior and medial frontal gyri in AD patients compared to HC [20]. Another fMRI study with HC, MCI, and AD groups demonstrated that the FC between the amygdala and the prefrontal cortex progressively decreased in patients (HC > AD-MCI > ADD) [23]. Our findings are consistent with these previous studies regarding the abnormal FC between the amygdala and frontal regions and sensorimotor network areas including the precentral gyrus. Unlike these previous studies, however, we demonstrated also a significant FC difference between the aMCI and SCI groups, where the SCI group consisted of individuals with memory complaints but no observable deficits in memory and other cognitive tests. Although we did not observe a significant amygdala FC difference in the ADD-aMCI comparison, the more extended FC disruption in the ADD-SCI comparison relative to the aMCI-SCI comparison suggests that there is a cumulative effect along the continuum from SCI to ADD on the FC impairment of the amygdala.

SCI represents an early stage of cognitive decline and can be associated with the early Braak stages (I-II) of AD [24]. According to Braak staging in AD, a small number of NFTs appear in the amygdala in Braak stage III and increase to moderate and large amounts by stages IV and V [1]. During Braak stages III-IV, NFTs significantly spread from the entorhinal cortex to the limbic regions, including the amygdala, which are stages associated with MCI [25]. Accordingly, our findings might be interpreted as the impact of NFT accumulation in the amygdala along the Braak stages on its connectivity.

Furthermore, strong correlations were obtained between the reduced FC of the left amygdala with frontal regions, fusiform gyrus, and the sensorimotor network regions that are precentral and postcentral gyri and the memory (FCSRT) and global cognitive screening (MMSE) scores. These findings are consistent with those reported by Yao et al. [21], who identified positive correlations between reduced FCs of the amygdala and prefrontal regions, the superior temporal gyrus (STG), and the precentral gyrus with MMSE scores and delayed recall performance on the Auditory Verbal Learning Test (AVLT). In our study, significant correlations were observed not only with delayed recall but also with immediate recall measures, including TFR and TR scores. This finding contrasts with Yao et al., who reported significant correlations exclusively with delayed recall and not with immediate recall. This discrepancy may be attributed to the FCSRT’s superior sensitivity compared to classical list-learning tasts such as AVLT.

Although functional studies exploring the relationship between the amygdala and memory or global cognition in ADD and MCI are relatively limited, structural MRI research has consistently shown that amygdala atrophy is closely linked to memory deficits and diminished global cognition, highlighting its pivotal role in these conditions [2, 4, 8–10]. For instance, Hari et al. [10] recently conducted a volumetric MRI study with the medial temporal lobe subregions on the data of a similar subject group as in the present study. Amygdala volumes of the AD, MCI, and SCI groups were positively correlated with the FCSRT-TFR scores, linking larger volumes to better recall ability. Another volumetric MRI study by Goerlich et al. [9] showed that reduced gray matter volume of the left amygdala was associated with impaired memory performance in MCI patients. These structural studies underscore the amygdala’s role in episodic memory and particularly emphasize the left amygdala’s involvement in memory functions. While previous structural studies support our findings, our analysis of functional data offers a mechanistic perspective, illustrating how the left amygdala, in coordination with specific brain regions, contributes to memory and cognitive processes in ADD and aMCI patients.

Memory impairment in AD is primarily characterized by hippocampal-type amnesia, resulting from encoding difficulties. Even when tasks incorporate supportive encoding strategies or rely on low-demand retrieval processes, such as cued recall or recognition, improvement remains minimal [26, 27]. This distinct memory profile differentiates AD-related deficits from those observed in conditions such as normal aging, frontotemporal dementia, or subcortical dementias, where memory impairments are primarily attributed to attention deficits or retrieval problems [28]. Several studies have underscored the critical role of the amygdala in memory encoding and consolidation processes [29–33]. The amygdala modulates the encoding and consolidation of memories of emotionally-laden experiences into long-term and remote memory. Both encoding and subsequent recall of emotionally arousing (either pleasant or unpleasant) experiences are more likely than the emotionally neutral ones [29]. Neuroimaging studies emphasize a reciprocal interaction between the hippocampus and amygdala during the encoding of emotional memories [30, 31, 34]. This relationship highlights the amygdala’s role in influencing explicit memory processes through its modulation of hippocampal activity [32, 35]. Moreover, the amygdala is considered to modulate episodic memory consolidation by facilitating gene expression, synaptic plasticity, and other information storage processes in its target regions, including the hippocampus [33, 36–39].

In our study, a strong correlation was observed between FCSRT-TFR scores and reduced FC of the left amygdala. The FCSRT-TFR score provides a direct measure of spontaneous recall ability by evaluating memory performance without external cues. Considering the amygdala’s established role in memory encoding and consolidation, this finding suggests that impaired connectivity within the amygdala may negatively influence these processes in AD, beginning at the MCI stage, and potentially contributing to the characteristic memory deficits of this condition. Similarly, both FCSRT-TR and delayed recall scores were also correlated with reduced FC of the left amygdala. Low total recall scores in AD and amnestic MCI, even with retrieval facilitation, point to a fundamental deficit in the storage of information [40]. Based on our findings and the underlying nature of memory impairment in AD, the correlation between reduced FC of the amygdala and memory scores primarily points to the dysfunctional amygdala’s negative impact on encoding and consolidation processes, which are known to be significantly compromised in ADD and MCI. On the other hand, relatively fewer studies suggested that the amygdala may also play a functional role in the retrieval process [41–43]. Although our findings align with the amygdala’s potential negative impact on encoding and consolidation, the correlation between the impaired cortical connectivity of the amygdala and overall memory performance in our study suggests that we cannot entirely rule out a potential negative influence on the retrieval process as well.

The current study additionally showed a significant positive correlation between the FC of the left amygdala with the fusiform gyrus and the BFRT scores. The fusiform gyrus contains a cortical region called the fusiform face area (FFA), which is specialized for recognizing and processing faces [44]. An fMRI study by Herrington et al. [12] using dynamic causal modeling has demonstrated bidirectional connectivity between the fusiform gyrus and the amygdala and indicated that these areas influence each other’s functions during face perception Several studies have shown impairments in both emotional and non-emotional face recognition in AD patients [45–47]. A task-based fMRI study has demonstrated functional alterations in the amygdala response to both neutral and emotional facial expressions in mild AD patients compared to elderly controls [48]. In our study, the disruption of the connectivity between the amygdala and fusiform gyrus is directly correlated with low performance in face recognition, which is a very solid finding suggesting a clear mechanism for the deteriorated face recognition in ADD.

The amygdala is a complex structure and consists of several nuclei, each with distinct functional roles; however, due to its small size, it is relatively difficult to analyze the FC of each subdivision using fMRI techniques. In the current study, we examined disruptions in FC in the amygdala as a whole, but future studies using ultra-high field fMRI, which enhances spatial resolution, may help to more specifically investigate functional impairment in different subregions of the amygdala in terms of disease severity.

Our study presents some limitations. First, our study sample size was small. We were able to demonstrate the difference between the ADD and SCI, and between the aMCI and SCI groups, but we could not observe a statistically significant difference between the ADD and aMCI groups. In future studies with larger samples, differences between these groups may also reach statistical significance, and the relationship between the amygdala and other cortical areas in the transition phase between aMCI and ADD may be more clearly revealed. Second, in this study, the diagnoses of the SCI, aMCI, and ADD groups were based on clinical diagnostic criteria without biomarker confirmation. The latest diagnostic criteria for Alzheimer’s disease, which are still considered within a research framework and have not yet been fully integrated into routine clinical practice, emphasize a biomarker-based approach and highlight the importance of incorporating biomarkers alongside clinical judgment to improve disease diagnosis and staging [49]. Notably, it has been reported before that amyloid pathology is present in 25.6% of individuals clinically diagnosed with SCI and 48.3% of those with MCI, as confirmed by PET or CSF evaluations, while such pathology is observed in nearly 90% of individuals with ADD [50]. Future research integrating clinical diagnostic criteria with biomarkers may facilitate the identification of more biologically homogeneous patient groups, thereby contributing to a deeper understanding of the role of amygdala functional connectivity across different clinical stages of AD.

## Conclusion

In this study, we observed that the left amygdala shows a progressive disruption in its functional connections, particularly with the frontal areas in aMCI and ADD, indicating that these changes may be an early neuroimaging marker of AD. We further identified that impaired connectivity of the left amygdala was strongly associated with memory impairment starting from the MCI stage onward. These data suggest that the amygdala’s impact on episodic memory impairment in AD has been evident from the early stages of the disease. Moreover, we demonstrated that impaired connectivity of the amygdala with the fusiform gyrus was directly correlated with decreased BFRT scores in ADD which provides important evidence that face recognition impairment in ADD is associated with impaired connectivity between the left amygdala and fusiform gyrus.

## Data Availability

Raw data used in this study are not publicly available to comply with local regulations and to protect individuals’ privacy in accordance with the Turkish Personal Data Protection Law.
